# Hypoxia and Extracellular Acidification as Drivers of Melanoma Progression and Drug Resistance

**DOI:** 10.3390/cells10040862

**Published:** 2021-04-09

**Authors:** Ewelina Dratkiewicz, Aleksandra Simiczyjew, Justyna Mazurkiewicz, Marcin Ziętek, Rafał Matkowski, Dorota Nowak

**Affiliations:** 1 Department of Cell Pathology, Faculty of Biotechnology, University of Wroclaw, Joliot-Curie 14a, 50-383 Wroclaw, Poland; aleksandra.simiczyjew@uwr.edu.pl (A.S.); justyna.mazurkiewicz2@uwr.edu.pl (J.M.); dorota.nowak@uwr.edu.pl (D.N.); 2 Department of Oncology and Division of Surgical Oncology, Wroclaw Medical University, Plac Hirszfelda 12, 53-413 Wroclaw, Poland; zietek.m@dco.com.pl (M.Z.); rafal.matkowski@umed.wroc.pl (R.M.); 3 Wroclaw Comprehensive Cancer Center, Plac Hirszfelda 12, 53-413 Wroclaw, Poland

**Keywords:** melanoma, hypoxia, acidification, drug resistance, tumor microenvironment, immune escape, invasiveness

## Abstract

Hypoxia and elevated extracellular acidification are prevalent features of solid tumors and they are often shown to facilitate cancer progression and drug resistance. In this review, we have compiled recent and most relevant research pertaining to the role of hypoxia and acidification in melanoma growth, invasiveness, and response to therapy. Melanoma represents a highly aggressive and heterogeneous type of skin cancer. Currently employed treatments, including BRAF V600E inhibitors and immune therapy, often are not effective due to a rapidly developing drug resistance. A variety of intracellular mechanisms impeding the treatment were discovered. However, the tumor microenvironment encompassing stromal and immune cells, extracellular matrix, and physicochemical conditions such as oxygen level or acidity, may also influence the therapy effectiveness. Hypoxia and acidification are able to reprogram the metabolism of melanoma cells, enhance their survival and invasiveness, as well as promote the immunosuppressive environment. For this reason, these physicochemical features of the melanoma niche and signaling pathways related to them emerge as potential therapeutic targets.

## 1. Introduction

Cutaneous melanoma is a very heterogeneous cancer characterized by pronounced invasive abilities and is responsible for 80% of deaths caused by skin cancer [1]. It arises from transformed neural crest-derived melanocytes, which, in physiological conditions upon UV exposure, produce the pigment melanin, and in cooperation with keratinocytes, they protect skin cells against UV-induced DNA damage [2].

While melanoma is recognized to exhibit high heterogeneity, a *BRAF V600E* mutation present in a gene encoding a member of the Ras/MAPK (mitogen-activated protein kinase) signaling pathway is a predominant genetic aberration detected in over 40% of melanoma patients [3,4]. Together with other specific markers, i.e., PD-L1 (programmed death ligand 1), CTLA-4 (cytotoxic T-lymphocyte-associated protein 4), this molecular feature of melanoma led to the development of targeted therapies, which improved the clinical results, so far relying on surgical excision and systemic chemo- and radiotherapy [5]. Nevertheless, melanoma continues to pose a great therapeutic challenge due to its rapidly emerging resistance to the majority of currently employed treatments [6].

Taking into account a great complexity of the melanoma microenvironment, it seems valid to consider specific factors of the tumor niche as potential therapy targets. Melanoma milieu consists of a number of cell types (e.g., keratinocytes, cancer-associated fibroblasts (CAFs), adipocytes, endothelial, and immune cells) and elements of extracellular matrix (ECM) [7]. It also exhibits a specific set of biophysical properties (e.g., hypoxia, acidification), which are able to influence the progression of cancer and its drug sensitivity [8,9]. In our previous works, we addressed the significance of crosstalk between cancer and stromal cells in melanoma growth, progression, and drug resistance [7,10]. Here, we endeavor to comprehensively review the most essential and recent research in the field of two closely related features of the tumor niche—hypoxia and acidification. We focus mainly on hypoxia- and acidification-induced aggressiveness and drug resistance of melanoma, as well as on novel anticancer treatment targeting these elements of the tumor microenvironment (TME).

## 2. Hypoxia

### 2.1. Molecular Basis of Hypoxia

**Figure 1:**
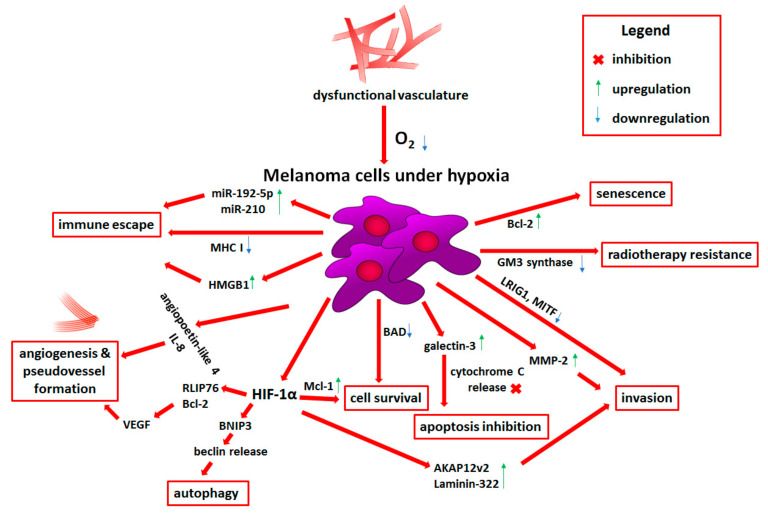
Influence of hypoxia on melanoma progression. Detailed descriptions can be found in the text. Abbreviations: miR, microRNA; MHC I, major histocompatibility complex class I; HMGB1, high mobility group box 1 protein; IL-8, interleukin 8; VEGF, vascular endothelial growth factor; RLIP76, Ral-interacting protein of 76 kDa; Bcl-2, B-cell CLL/lymphoma 2; BNIP3, Bcl-2 interacting protein 3; HIF-1α, hypoxia-inducible factor 1 α; Mcl-1, myeloid cell leukemia 1; BAD, Bcl-2-associated agonist of cell death; AKAP12v2, A-kinase anchor protein 12 variant 2; MMP2, matrix metalloproteinase 2; LRIG1, leucine-rich repeats and Ig-like domains 1; MITF, microphthalmia-associated transcription factor; GM-3, monosialodihexosylganglioside.

A prevalent feature of solid tumors is a state of hypoxia, characterized by a low oxygen level (<1%), in comparison to a physiological O_2_ concentration, reaching approximately 4–10%, varying between different types of tissues and organs [11,12]. This pathological condition is a result of a dysfunctional vasculature developed upon the cancer cells’ stimulatory signals, which is unable to provide a sufficient supply of oxygen to the intensively growing tumor mass, especially in the case of solid tumors (Figure 1) [13]. For skin tissue, the values of O_2_ concentration vary from 17.7% for the outer layers of the epidermis and down to trace concentrations for some areas of the dermis [14]. While the response of cells to hypoxia is strongly dependent on the tissue type and its initial oxygenation, the lack of O_2_ often leads to the cell apoptosis. Considering the adaptation of cancer cells to adverse conditions, paradoxically, hypoxia frequently supports their survival and more aggressive phenotype [11,15]. The data acquired from melanoma cancer patients indicate the positive correlation between the presence of hypoxia within the tumor mass and a poor prognosis for these patients [16].

The master regulators of a hypoxic response are proteins called hypoxia-inducible factors (HIF). The active protein capable of binding to the hypoxia response elements in the DNA sequence consists of two subunits—HIF-α and HIF-β [17]. HIF-1β is a constitutively expressed protein located in the nucleus, while the expression level of three members of the HIF-α family (HIF-1α, HIF-2α, and HIF-3α) is highly dependent on the cellular oxygen concentration [18,19]. In the presence of O_2_, the PHD (prolyl hydroxylase domain protein) enzymes hydroxylate HIF-α molecules, which leads to their ubiquitination by the ubiquitin ligase complex recruited by the pVHL protein (von Hippel–Lindau tumor suppressor) and subsequent degradation in the proteasome. The absence of oxygen inhibits PHDs activity, promoting the stability of HIF-α, as well as allowing for its translocation to the nucleus and dimerization with HIF-1β [20,21].

HIF-1α can be also activated in hypoxia-independent manner in response to growth factors and cytokines stimulation, which activate signaling pathways such as Ras/MAPK, PI3K (phosphoinositide 3-kinase)/AKT or NF-κB (nuclear factor-kappa B), known to be often upregulated in cancer cells [22,23]. Additionally, epigenetic changes and mutations may influence the level and stability of this transcription factor [24].

The overexpression of HIF-1α was detected in biopsies derived from patients suffering from skin cancer and uveal melanoma. It was also elevated in metastatic tissue compared to the primary tumors as well as correlated with high expression of proliferative and vascular markers [25,26]. A number of studies report the upregulation of HIF-1α expression in a panel of primary and metastatic melanoma cell lines even in experimental normoxic conditions, which can be partially attributed to the slightly hypoxic conditions present in the normal skin tissue, especially in the lower layers of the epidermis where the melanocytes reside [14,27,28,29].

On the other hand, the role of HIF-2α in melanoma was not as extensively studied and the data are often inconclusive. A positive correlation between poor prognosis and the high expression of HIF-2α as well as VEGF (vascular endothelial growth factor) was observed in samples isolated from nodular malignant melanomas of the skin, while Hao et al. showed that HIF-2α overexpression induced the stemness in melanoma cells through inhibition of p21 [30]. However, another study indicated that the HIF-2α in a melanoma cell line model led to the reduction of these invasive abilities [31,32]. Interestingly, it was also shown that HIF-2α-deficient tumors exhibit increased interferon-based signaling, antigen presentation, as well as CD8+ T cell infiltration and activation [33]. These differences in HIF-2α-induced cellular responses may be cell- and organ-specific or stem from the temporary balance between different HIF isoforms. Less is known about the HIF-3α level in skin cancers.

The expression of HIF-α regulating enzymes may also influence tumor aggressiveness. While hypoxia inhibits the activation of PHD2 due to the lack of oxygen required for its function, melanoma cells were shown to lose the expression of this enzyme during transition from benign nevi to melanoma. Moreover, the simultaneous presence of *BRAF V600E* mutation and loss of PHD2 expression in melanocytes led to their malignant transformation towards highly invasive melanoma [34].

### 2.2. Role of Hypoxia in Melanoma Progression

#### 2.2.1. Prosurvival Role of Hypoxia

Upon the introduction of hypoxic conditions, apoptotic pathways are induced in the majority of normal cells and a population of cancer cells, which eventually leads to cell death. However, cancer cells often exhibit HIF-1α stable expression and activation, which renders them resistant to the adverse microenvironment [35].

The hypoxic response may be mediated by small non-coding microRNAs (miRs), which post-transcriptionally regulate the expression of various genes. It was shown that miR-211 is able to sensitize melanoma cells to hypoxia-induced cell death, however cancer cells lose its expression during melanomagenesis, especially in hypoxic conditions [36]. Also, the upregulation of miR-302 elicits a hypoxia-related decrease in the viability of melanoma cells [37].

One of the main regulators of hypoxia-induced apoptosis is the tumor suppressor protein, p53. Leszczynska et al. identified a group of p53 proapoptotic target genes (e.g., *INPP5D* (inositol polyphosphate-5-phosphatase), *CYFIP2* (cytoplasmic FMR1-interacting protein 2)), which, upon expression, inhibit AKT activity leading to cell death. Interestingly, downregulation of these genes, presumably as a result of hypoxia-induced mutations in the gene encoding p53, was detected in melanoma patients as well as correlated with worse prognosis [38].

It was also shown that following hypoxia exposure, melanoma cells bearing *BRAF V600E* mutation exhibit downregulation in the expression of genes encoding apoptotic regulators—*BAD* (Bcl-2-associated agonist of cell death) and *BCL2L1* (Bcl-2-like 1, also known as Bcl-X) [39]. While a decrease in *BAD* expression may act antiapoptotic and promote cell survival (Figure 1), the data concerning *BCL2L1* could be interpreted disparately, as the gene can give rise to two isoforms that display opposite functions [40]. The HIF-1α-induced overexpression of antiapoptotic protein Mcl-1 (myeloid cell leukemia 1) was also observed in BRAF-mutated melanoma cells (Figure 1). It correlated with a higher rate of resistance to anoikis, which is a form of apoptosis resulting from lack of adhesion. This ability is crucial for malignant cells to survive outside of the primary tumor site [41,42].

Furthermore, using in vitro murine melanoma model, Bacchi et al. have shown that cells exposed to hypoxia and high glucose concentration exhibit upregulated expression of galectin-3, which displays some similarities to Bcl-2 (B-cell CLL/lymphoma 2) family proteins and is also able to block apoptosis (Figure 1) [43]. However, others have reported that galectin-3 may act as a metastasis suppressor or even sensitize BRAF-mutated melanoma cells to vemurafenib (BRAF V600E inhibitor) treatment [44,45].

Additionally, Wang et al. showed that activation of hypoxic response involves overexpression of Bcl-2, which leads to a state of permanent cell arrest, so-called senescence (Figure 1) [46]. Although senescent cells do not proliferate, they still exhibit metabolic activity and are able to influence adjacent cells present in the tumor microenvironment. Hypoxia-induced secretion of MMP2 (matrix metalloproteinase 2) by senescent cells was also implicated in facilitating the invasion of melanoma cells (Figure 1) [47].

The levels of lipid raft-associated gangliosides as well as ganglioside synthesizing and processing enzymes, which are involved in the regulation of endoplasmic reticulum stress response and apoptosis, can also be influenced by hypoxia. Low oxygen levels led to the downregulation of GM3 (monosialodihexosylganglioside) synthase in melanoma cells, which produces a common precursor of gangliosides. Reduction in GM3 levels allows for elevated receptor tyrosine kinase dimerization and subsequent hyperactivation of ERK (extracellular signal-regulated kinase) kinase. This process contributed to the emergence of radiotherapy resistance, taking into account the antiapoptotic and cell cycle arrest-inducing role of ERK (Figure 1) [48,49].

Finally, cancer cells exposed to hypoxia can induce autophagy, a process responsible for maintaining cell homeostasis in a lysosome-mediated way. Activation of HIF-1α stimulates BNIP3 (Bcl-2 interacting protein 3), which in turn releases beclin from its association with Bcl-2 or Bcl-XL and leads to autophagy initiation (Figure 1) [50]. Increased level of BNIP3 was detected in melanoma cells under hypoxia, which also correlated with reduced response to anti-PD-1 (programmed cell death 1) treatment [51]. The overexpression of beclin 1 was also associated with highly pigmented and hypoxic areas of uveal melanoma tumors, which characterized patients with poor prognosis [52]. However, prolonged autophagy induced by nutrients deprivation, together with hypoxic conditions, may eventually lead to cell death [53].

#### 2.2.2. Angiogenesis and Vasculogenic Mimicry Induced by Low Oxygen Levels

Hypoxia arises as a consequence of insufficient or dysfunctional vasculature in the area of intensively proliferating cancer cells. To avoid growth deceleration or even necrosis, cells trigger HIF-1-dependent pathways, which leads to the initiation of angiogenesis, where new vessels are formed from preexisting vascular networks, or results in the emergence of cancer cells-derived pseudovessels in a process called vasculogenic mimicry [54].

The main component of blood vessels are endothelial cells (ECs), which form a barrier between the conducted physiological fluids and the tissue. New vessels can be formed from preexisting ones under proangiogenic stimuli including gradient of VEGFA (vascular endothelial growth factor A), which, together with other factors, directs the vascular sprouting of motile ECs [55]. Das et al. reported the importance of communication between melanoma and endothelial cells for the survival of ECs in adverse conditions. Following exposure to melanoma conditioned medium (CM), the ECs were able to endure long-term hypoxia, while a similar result was not observed for melanocytes-derived CM. The activation of AKT and MAPK/ERK pathways was responsible for this effect [56]. It was also shown that, while mature ECs form vessel-like tubes, melanoma cells preferentially recruit ECs early progenitors to build pathological vasculature. This process is mediated by hypoxia-induced stabilization of cancer stem-like cells (CSCs), which exhibit increased activity of one of the CSC markers—ALDH (aldehyde dehydrogenase) [57].

Mutual regulation between melanoma cells and ECs under hypoxic conditions also involves functional regulators of HIF-1, which influence the angiogenic components of the cancer cell secretome including endothelin-1 and RLIP76 (Ral-interacting protein of 76 kDa) signaling, which in turn activate HIF-mediated VEGFA and VEGFC expression. Moreover, melanoma cells treated with conditioned medium derived from ECs were more prone to form vessel-like channels (Figure 1) [58,59]. Another protein involved in the regulation of endothelial cell tubes formation through the activation of VEGFA expression is DRG2 (developmentally regulated GTP-binding protein 2), which participates in the HIF-1α translocation to the nucleus. It was also shown that DRG2 expression correlates with poor survival of melanoma patients and was more pronounced in metastatic compared to primary tumors [60]. Interestingly, previously mentioned antiapoptotic protein Bcl-2, following its relocation to the nucleus, is also able to induce VEGF expression in HIF-1-dependent way [61].

Elevated expression of VEGF and HIF-1α in correlation with vascularity in uveal melanoma patients was also observed. Moreover, hypoxic uveal melanoma cells exhibited increased angiopoietin-like 4 expression, which inhibition in vitro and in vivo reduced angiogenic potential of these cells (Figure 1) [62]. VEGF expression was also detected along vessel-like channels in specimens from choroidal melanoma patients, which originated as a result of the vasculogenic mimicry. Hypoxia was responsible for VEGF expression upregulation and subsequently for PI3K/AKT-mediated development of pseudovessels [63].

High level of another proangiogenic molecule, IL-8 (interleukin 8), was detected in samples collected from melanoma patients representing different stages of cancer (Figure 1). Additionally, upregulation of IL-8 correlated with the elevated expression of multicellular (e.g., endothelial and hematopoietic stem cells) CD34 antigen, and together they were associated with worse prognosis [64]. Similarly, Timani et al. linked hypoxia-driven IL-8 upregulation and poor prognosis of melanoma patients to reduced expression of a multifaceted nuclear protein, Tip110 [65]. Elevated level of IL-8 and VEGF following GRM1 (metabotropic glutamate receptor 1)-dependent activation of AKT/mTOR (mechanistic target of rapamycin)/HIF-1 axis was also found in media derived from melanoma cells, while inhibition of GRM1 in melanoma patients led to the reduction of vasculature density in tumor specimens [66]. Melanoma cells, however, are not the only source of proangiogenic factors in the tumor microenvironment (TME). Activated fibroblasts (also called CAFs), which may constitute even 80% of the tumor mass, when exposed to hypoxia, also secrete elevated level of VEGFA and IL-6 compared to the cells cultured in normoxia [67,68].

#### 2.2.3. Melanoma Plasticity and Invasiveness under Hypoxia

Hypoxia has been repeatedly correlated with highly aggressive cancer phenotype and worse prognosis for patients [15]. This aggressiveness is often associated with elevated plasticity of tumors, which exhibit potent invasive abilities.

The master regulator of melanocyte development and a more proliferative cancer phenotype is *MITF* (microphthalmia-associated transcription factor), which expression is distinctive for melanoma-initiating cells. Its downregulation is often indicative of transcriptional reprogramming resulting in the emergence of a migratory phenotype [69,70]. Indeed, hypoxia exposure of melanocytes harboring *KIT* mutations led to the reduction of MITF expression and their malignant transformation (Figure 1) [71]. Widmer et al. also noted a loss of melanocytic markers in melanoma biopsies, which correlated with hypoxic regions [72]. Furthermore, melanocytes exposed to low oxygen levels underwent downregulation of anti-metastatic and antiangiogenic molecule—PEDF (pigment epithelium-derived factor). Loss of PEDF expression was associated with the acquisition of an invasive phenotype by transformed cells [73].

Hypoxia may also induce the selection of cancer stem-like cells, which exhibit a self-renewal ability, increased invasiveness, and elevated drug resistance. Nodal, one of the regulators of cell stemness, was shown to be induced following hypoxia exposure, and its increased expression correlated with a more aggressive and resistant melanoma phenotype. Inhibition of Nodal expression partially sensitizes cells to chemotherapy, confirming its role in the development of drug resistance [74].

High aggressiveness of melanoma cells may also result from the induction of epithelial-to-mesenchymal transition (EMT), during which cells lose their epithelial polarity and intercellular junctions in favor of a mesenchymal phenotype allowing them to invade through the tissues. It was shown that hypoxia reduced the expression of LRIG1 (leucine-rich repeats and Ig-like domains protein 1), a negative regulator of ERBB receptors, which led to the activation of EGFR (epithelial growth factor receptor)/ERK signaling and induction of EMT (Figure 1). It was also accompanied by an elevated rate of migration, invasion, and vasculogenic mimicry. Erlotinib, a specific inhibitor of EGFR, was able to abrogate invasive abilities of LRIG1-devoid cells under hypoxia, of which observation may help in the development of anti-melanoma therapies [75].

EMT process, as well as ERK/AKT-mediated invasion, can be activated by Notch signaling, which is also promoted by low oxygen level [76,77]. Liu et al. indicated that the hypoxic response is mediated by Snail1, one of the main transcriptional regulators of EMT. Hypoxia led to the elevated expression of mesenchymal markers (Snail1, N-cadherin (neuronal cadherin), α-SMA (α-smooth muscle actin), SOX10 (SRY-box transcription factor 10)) with simultaneous reduction in E-cadherin (epithelial cadherin) level in melanoma cells. Additionally, cells exposed to low oxygen concentration demonstrated higher resistance to chemotherapy, including cisplatin and temozolomid treatment [78].

Increased invasiveness of melanoma cells was also reported following treatment with conditioned media derived from activated fibroblasts cultured in hypoxia [68,79]. This process could be mediated by molecules secreted by CAFs—SDF-1 (stromal cell-derived factor-1) and IL-6. SDF-1 was also shown to induce EMT in uveal melanoma cells, while IL-6 is able to promote cell motility through upregulation of WNT5A (wingless-type mammary tumor virus integration site family member 5A) expression [68,80,81].

Several reports also indicate the role of hypoxia in the expression of cytoskeleton regulating proteins and subsequently in melanoma invasive abilities. The formation of migratory protrusions and focal adhesions is governed by prominent members of the Rho GTPase family—RhoA (Ras homolog family member A), Rac1 (Rac family small GTPase 1), and Cdc42 (cell division cycle 42) [82]. An elevated HIF-1α level was associated with increased expression and activation of RhoA, as well as higher migratory potential of HT168-M1 melanoma cell line [83]. Stimulation of another member of the small GTPase family, Rab5, which is involved in focal adhesion dynamics, migration, and Rac1 activation, was also facilitated by low oxygen levels. Hypoxia promoted re-localization of Rab5 to the cell leading edge and its association with focal adhesions, as well as FAK (focal adhesion kinase) phosphorylation and hypoxia-driven melanoma cell migration [84].

The transduction of signals between the cell surface and cytoskeleton is regulated by scaffold proteins which act as a binding-platform for kinases and other actin-associated proteins. HIF-1α-induced expression of AKAP12v2 (A-kinase anchor protein 12 variant 2) was shown to promote migration and invasion of melanoma cells (Figure 1) [85]. Annexin A3 also plays a role in actin-based signal transduction. Xu et al. noted an elevated level of this protein in melanoma patients, while downregulation of annexin A3 reduced migratory abilities of melanoma cells in HIF-1α/VEGF-dependent manner [86].

The interaction of cancer cells with the extracellular matrix is essential in a number of processes, including adhesion, migration, and invasion. This interplay is governed by surface proteins that act as receptors for soluble ligands or as scaffolds for the ECM molecules present in the extracellular space [87]. Dahl et al. reported that the exposure to hypoxia was responsible for elevated level of α_V_β_3_ integrin and increased adhesion to vitronectin in a metastatic murine melanoma model [88]. It was also demonstrated that HIF-1α is able to upregulate the expression of laminin-322, one of the main ECM elements secreted by keratinocytes, which was shown to promote adhesion and migration of melanoma cells (Figure 1) [89,90]. There are reports indicating that syndecans, proteoglycans responsible a.o. for the ECM organization, are involved in the dysregulation of the TME. CAFs exhibited an elevated level of syndecan-4, while high expression of syndecan-3 was detected in melanoma cells, epithelial cells, and tumor-associated macrophages, which also correlated with hypoxia gene signature [91]. Finally, hypoxia is known to upregulate LOX (lysyl oxidase), one of the enzymes responsible for collagen crosslinking. LOX activity was shown to promote cancer metastasis and formation of pre-metastatic niches [76,87].

Cancer invasiveness can be further regulated by microRNAs. Downregulation of several miRs (e.g., miR-224-5p, miR-23a-5p, mi-23b-5p) with simultaneous upregulation of miR-1290 levels correlated with elevated invasive abilities of melanoma cells [92,93,94]. In the case of melanoma, the exact effect of FXR2 (fragile X mental retardation syndrome-related protein 2) expression regulation by miR-1290 is still poorly understood [95]. However, it was shown that the depletion of FXR2 homolog—FXR1—in melanoma led to impaired invasion and metastasis [96,97].

#### 2.2.4. Metabolic Reprogramming Induced by Low Oxygen Level

The principal metabolic strategy of human cells relies on oxygen. To produce energy, cells employ so called oxidative phosphorylation (OXPHOS), which takes place in the mitochondria. Hypoxic conditions are responsible for metabolic reprogramming of cancer cells, which start to favor glycolysis and subsequent lactic fermentation, a shift also characterizing Warburg effect [98].

Koch et al. showed that melanoma cells exposed to low oxygen concentration exhibited a highly glycolytic phenotype, including elevated expression of GLUT1 (glucose transporter isoform 1), HK2 (hexokinase 2), and LDH-A (lactate dehydrogenase A). Furthermore, BRAF-mutated cells demonstrated increased GLUT1 levels and invasive abilities in hypoxic conditions [99]. Another enzyme involved in glycolysis, PFKFB4 (6-phosphofructo-2-kinase/fructose-2,6-biphosphatase 4), was also demonstrated to be upregulated following exposure to hypoxia and its high expression correlated with poor prognosis of melanoma patients [100]. Zhuo et al. have also observed upregulation of GLUT1 and HK2 expression, as well as elevated lactate production and reduced mitochondrial content consistent with cancer cells glycolytic shift in a melanoma model in vitro. Interestingly, they also noted decreased sensitivity of the examined cell lines to cisplatin and doxorubicin upon hypoxia exposure [101].

Increased activity of LDH-A also leads to a change in metabolites’ balance—there is less α-ketoglutarate (α-KG) produced than L-2-hydroxyglutarate. On the one hand, together with the low supply of glutamine in the tumor microenvironment, which is essential for α-KG production, it leads to the reduced activity of demethylases using α-KG as a co-substrate. On the other hand, L-2-hydroxyglutarate may act as an inhibitor of these enzymes. Low activity of demethylases results in accumulation of aberrations in the methylation of histones (e.g., H3K27me3), DNA, and RNA, further inducing drug resistance and heterogeneity of patient-derived BRAF-mutated melanoma cells [102,103].

Another regulator of decreased mitochondrial metabolism is pyruvate dehydrogenase kinase 4 (PDK4). It phosphorylates pyruvate dehydrogenase, which in turn leads to reduced OXPHOS, and thus lowered oxygen use, resulting in HIF-1α stabilization. *PDK4* is a target gene of miR-211, which expression is lost during melanoma malignant transformation. This small non-coding RNA acts as a metabolic switch, allowing cancer cells to survive during hypoxia [36].

The tricarboxylic acid (TCA) cycle, which in normoxia is tightly linked to glycolysis through the production of acetyl-CoA from pyruvate, in hypoxic conditions starts to run in reverse to maintain cell proliferation. In this case, glutamine acts as a main carbon source for acetyl-CoA, citrate, and fatty acids. Additionally, BRAF- and NRAS-mutated melanoma cells exhibit elevated utilization of this amino acid as a substrate for fatty acid biosynthesis [104]. One of the TCA cycle intermediates, succinate, revealed its role as a HIF-1α stabilizing agent, as it inhibits PHD2, an enzyme responsible for HIF-1α degradation. Interestingly, this process is partially regulated by MITF [105].

#### 2.2.5. Hypoxia-Dependent Immune Escape

Cancer cells also utilize survival strategies, which rely on suppression of the organism immune response against malignantly transformed cells. Hypoxia may enhance the antitumor immune cell function. However, usually a low oxygen level in the tumor niche promotes recruitment of immunosuppressive cells (e.g., Tregs (T regulatory cells), MDSCs (myeloid-derived suppressor cells), M2 macrophages) and secretion of proinflammatory cytokines (e.g., IL-10), as well as leads to reduced cytotoxic activity of NK (natural killer) and T cells [106,107].

CD8+ cytotoxic T cells (CTLs), upon recognition of antigen presented by other cells, induce cancer cell death via release of granzymes, perforin, etc. [108]. In the case of tumors, to accomplish their immunogenic task, CTLs need to leave the vasculature and come in direct contact with cancer cells. Indeed, the accumulation of CD8+ cells occurred around the peripheral blood vessels in the samples derived from melanoma patients’ biopsies. Additionally, reduced number of tumor-infiltrating CTLs correlated with a worse prognosis. In vitro studies have shown that the diminished motility of immune cells towards the tumor mass may result from hypoxia and decreased rate of OXPHOS [109]. Marijt et al. observed that hypoxia together with glucose-deprivation impaired antigen presentation via MHC (major histocompatibility complex) class I and subsequent recognition by CTLs, even in the presence of the stimulatory cytokine IFNγ (interferon γ) [110]. Loss of sensitivity to IFN treatment as a result of HIF-1α/RIG-I (retinoic acid-inducible gene I)-dependent downregulation of IFNαR (interferon α receptor) expression and consequent diminished NK- and CTL-mediated cell death was also observed in human and murine melanoma cells [111].

Reduced cytotoxic activity of CTLs against cancer cells could also stem from HIF-1α-driven decreased expression of MHC class I on the surface of tumor cells, as well as be associated with tumor-protective role of extracellular adenosine (Figure 1). Hypoxia was shown to upregulate the expression of adenosine producing enzymes (CD39, CD73), which in turn contribute to elevated adenosine and adenosine receptor levels, followed by activation of CREB (cAMP-response element binding protein)-mediated immune suppression via proinflammatory molecules expression (e.g., IL-2, IL-6, IL-10) [112,113]. The adenosine-induced signaling may result in inhibition of macrophage activation and NK cell infiltration, as well as severely changed T cell profiles. It leads to the reduction of antitumoral effector T cell proliferation, maturation, terminal differentiation, migration, and finally their cytotoxic activity, with a simultaneous increase in T cell switch towards an immunosuppressive Tregs lineage, partially supported by an upregulation in CTLA-4 expression [114,115].

Suppression of CTL cytotoxic activity may be also mediated by miR-192-5p produced by melanoma cells under hypoxic conditions, which is then transported to immune cells through connexin-43-constituting gap junctions of cancer cells [116]. Moreover, hypoxia-induced miR-210 expression disturbs melanoma sensitivity to T cell lysis, through the inhibition of genes known to influence this process (*PTPN1* (protein tyrosine phosphatase non-receptor type 1), *HOXA1* (homeobox A1), *TP53I11* (tumor protein P53-inducible protein 11)) (Figure 1) [117,118].

On the other hand, Kajiwara et al. noted that murine melanoma cells pre-incubated in hypoxia elicit greater antigen-specific CTL response compared to cells growing in normoxia. This process was facilitated by upregulation of MHC class I mediated by ERO1α (endoplasmic reticulum oxidoreductase 1α) activity, one of the enzymes responsible for the formation of disulfide bonds in the polypeptide chain [119]. Finally, CTLs used in anticancer therapy are usually cultured in normoxia, while the targeted tumor resides in hypoxic conditions. It was shown that pre-incubation of CTLs in hypoxia improves their ability to kill malignant cells, possibly due to minor changes in the composition of the cytotoxic granules [120].

Stress-related catecholamines, i.e., adrenaline and noradrenaline, which interact with β-adrenoreceptors, were also shown to promote melanoma progression and metastasis. Following exposure to hypoxia, the upregulation of β_3_-adrenoreceptors was noted in immunosuppressive peripheral blood mononuclear cells (NK cells, MDSCs, and Tregs). Application of β-blockers (e.g., propranolol) led to the attenuation of immunosuppressive cell subpopulations, as well as improved the number of cytotoxic immune cells (NK, CTLs) within the TME. Inhibition of hypoxia-induced β_3_-adrenoreceptors also promoted M1 polarization of macrophages, reducing the rate of M2 type, a phenotypic shift associated with immune suppression [121].

The ability of cancer cells to recruit immunosuppressive cells or re-educate them towards pro-tumoral phenotype is often dependent on paracrine signaling. For instance, uveal melanoma cells exposed to hypoxia produced an assortment of proinflammatory molecules (e.g., plasminogen, TGFβ (transforming growth factor β), endothelin 1) and the tumor cell-derived conditioned medium was able to inhibit the differentiation of dendritic cells [122]. The recruitment of M2 macrophages in a hypoxia-dependent manner was observed in VEGF- and HMGB1 (high mobility group box 1)-secreting melanoma cells (Figure 1) [123,124]. Stimulated M2 macrophages subsequently generated great amounts of proinflammatory IL-10. Furthermore, melanoma patients with diagnosed metastatic tumor exhibited elevated serum concentration of HMGB1 [124]. Yang et al. have shown that low oxygen concentration in the tumor microenvironment leads to downregulation of MHC class II expression in tumor-associated macrophages, priming their shift towards an M2 phenotype [125].

#### 2.2.6. Reduced Therapy Efficiency and Novel Hypoxia-Targeted Treatment Strategies

The adaptive mechanisms that cancer cells employ to survive and even thrive in prolonged hypoxia, often lead to the emergence of drug resistance. Gaustad et al. have indicated that, following surgical excision of the tumor, chemo- or radiotherapy alone may not be sufficient considering that even diminutive tumors display the presence of hypoxic regions stemming from disrupted vascular function [126]. Moreover, if the radiation treatment is not continuous, it may lead to even faster repopulation of tumor cells. This phenomenon is caused by the re-oxygenation of hypoxic cells stemming from the decreased oxygen demand exhibited by dying cancer cells [127]. There are several possible mechanisms for chemo- and radiotherapy failure in the case of hypoxic tumor. Firstly, following HIF-dependent growth deceleration and inhibition of DNA replication, the DNA crosslinking or intercalating compounds have worse access to the tightly packed genetic material. Secondly, upon metabolic shift, which limits the activity of mitochondria, these organelles are less prone to mitochondrial DNA damage [101]. Furthermore, the lack of oxygen leads to the reduction in therapy-induced ROS (reactive oxygen species) levels, which are the mediators of irradiation-driven DNA damage [128]. Finally, hypoxia response elements are able to induce prosurvival pathways, mainly due to a loss of sensitivity to p53-mediated cell apoptosis by cancer cells [129].

For this reason, a plethora of novel therapeutic strategies targeting the hypoxic tumor microenvironment are being introduced to anti-melanoma treatment. They can be categorized into several groups of drugs, which target hypoxia indirectly (e.g., antiangiogenic molecules) or directly (e.g., inhibitors of HIF dimerization, nanoplatforms delivering oxygen) (Table 1).

Taking into account the importance of angiogenesis in tumor development, a great number of therapeutic strategies targeting proangiogenic pathways in anti-melanoma treatment were introduced, including anti-VEGF monoclonal antibodies (e.g., bevacizumab) or multikinase inhibitors (Table 1) [130]. Another approach to block VEGF is using a soluble form of VEGF receptor (sVEGFR), which neutralizes this growth factor. It was proposed as an adjuvant therapy allowing for vascular normalization using a sVEGFR vector under a hypoxia-inducible promotor [131]. Furthermore, GM-CSF (granulocyte-macrophage colony-stimulating factor), a cytokine secreted by fibroblasts, immune and endothelial cells, was able to shift macrophage production of VEGF towards sVEGFR1 in hypoxic conditions, which resulted in reduction of tumor mass (Table 1). These findings also suggest the use of GM-CSF intratumorally to limit melanoma growth [132]. However, it was shown that even in combination with chemotherapy these drugs were not able to significantly reduce tumor growth and metastasis in the long term [130]. Yura et al. observed that the inhibition of endothelial cell-dependent vascularization may even accelerate cancer progression due to the hypoxia-induced process of vasculogenic mimicry [146].

However, novel ways to normalize the tumor vasculature, rather than abrogate it, may improve overall therapeutic effect. Agrawal et al. developed Sac-1004, a compound potentiating endothelial cell junctions mediated by VE-cadherin (vascular endothelial cadherin) via cAMP (cyclic adenosine monophosphate)/Rac/cortactin pathway (Table 1). It enhances cell-cell contact integrity and reduces vascular leakage. Sac-1004 monotherapy significantly inhibited tumor metastasis, EMT signature, as well as resulted in a substantial drop in the cancer stem-like cell subpopulation. As a combination therapy with a chemotherapeutic—cisplatin, this novel compound synergistically induced melanoma cell apoptosis [133].

Owing to their critical involvement as mediators of the hypoxic response, HIF proteins appear to be promising therapy targets. Indeed, acriflavine, an inhibitor of HIF dimerization, was shown to block melanoma metabolism and proliferation even in normoxic conditions in cells expressing HIF irrespective of microenvironment oxygenation (Table 1) [134]. Radioresistant melanoma cell line was resensitized following treatment with HIF-1α inhibitor—2-methoxyestradiol (Table 1). Moreover, resistant cells characterized by high expression of GLUT1 and LDH-A underwent partial suppression of their glycolytic state upon drug administration [135].

Cinnamaldehyde, a dietary flavonoid, was shown to block HIF accumulation in a PI3K/AKT/mTOR-dependent way (Table 1). Melanoma cells treated with this compound displayed reduced expression of VEGF, its receptor, MMP2/9, and markers of EMT, as well as decreased rates of invasive abilities and angiogenic activity [136]. Another drug, arylsulfonamide (64B), impaired the interaction between HIF-1α and its nuclear co-factors p300/CBP (CREB-binding protein), thus diminishing the p300 recruitment to the *CXCR4* (C-X-C chemokine receptor type 4) and *MET* (hepatocyte growth factor receptor) gene promoters and subsequent expression of these genes (Table 1). Uveal melanoma tumors treated with 64B exhibited decreased growth and rate of metastasis [147].

Currently, more complex therapeutic strategies, e.g., incorporating several active compounds and targeting molecules, are being introduced into the anticancer treatment. Alleviation of hypoxic conditions through the inhibition of oxygen consumption by cancer cells using metformin, was shown to improve the efficiency of anti-PD-1 immunotherapy (Table 1) [137]. Jiang et al. proposed combination therapy consisting of co-encapsulated doxorubicine and hemoglobin to deliver oxygen and mitigate hypoxia, followed by radiotherapy. The treatment sufficiently blocked the in vitro migration of melanoma cell and inhibited tumor growth in a mouse model (Table 1) [148]. Multifunctional immunoliposome CAT@aPDL1-SSL delivers the oxygen generating enzyme—catalase to the hypoxic regions of the tumor and reduces the extracellular acidity (Table 1). The delivery is driven by interaction between modified aPDL1 on the immunoliposome and PD-1 on the surface of the cancer cells. Using murine melanoma model, Hei et al. have shown improved activation and tumor infiltration by CD8+ CTLs upon mice treatment, and their subsequent prolonged survival [139].

The biomimetic nanoplatforms (mZCD and Au@MTM-HA) developed by two research groups combine multiple functionalities (Table 1). They deliver oxygen-generating molecules and inhibitors of ROS-scavenging antioxidants or chemotherapeutics. Additionally, the platforms include surface modifications to improve drug targeting. Using murine melanoma model, the effectiveness of mZCD treatment in combination with anti-PD-1 immunotherapy, as well as monotherapy with Au@MTM-HA was confirmed [140,141].

A class of prodrugs activated in hypoxic conditions was also tested in the case of melanoma. While monotherapy with tirapazamine, a DNA-damaging compound, failed to elicit a significant anticancer response, promising results were obtained using combination therapy with NTP (non-thermal plasma), also called ‘cold plasma’ (Table 1). NTP alone strongly induced ROS production and subsequent apoptotic response, whereas together with tirapazamine it displayed even higher effectiveness, resulting in 90% reduction in tumor volume in a murine melanoma model [142]. Another hypoxia-activated prodrug with DNA-alkylating activity, TH-302, also reduced tumor volume in combination with a standard chemotherapeutic (temozolimide) or inhibitor of multiple receptor tyrosine kinases (sunitinib), especially, when melanoma cells were first pre-treated with hypoxia-targeting drug (Table 1) [143,149].

An interesting approach to hypoxia-targeted drugs involves utilizing bacteria as drug delivery vectors. Liu et al. constructed a hypoxia-inducible vector expressing SPRY1/2 (sprouty RTK signaling antagonist 1/2), a negative regulator of receptor tyrosine kinase signaling, which was delivered to melanoma cells using an attenuated strain of *Salmonella typhimurium* (Table 1). They observed significant reduction in cancer cell proliferation mediated by inhibition of ERK phosphorylation [144]. A highly oxygen sensitive strain of the anaerobic bacterium *Clostridium novyi-NT* was also tested in hypoxic solid tumors including melanoma (Table 1). This strategy specifically targets the hypoxic and necrotic areas of tumors, induces oncolysis and immune response, and thus may be able to overcome the limitations of conventional anticancer therapy [145].

## 3. Acidification

### 3.1. Molecular Basis of Tumor Microenvironment Acidification

Another physicochemical factor that significantly affects the functioning of melanoma cells is tumor niche acidification. Although cancer cells produce excessive amounts of acidic molecules, the intracellular pH remains neutral or even basic [150]. This state is obtained through the activity of transporters and channels related to pH regulation, which export acid molecules outside [151]. However, buffer capacity of the tumor microenvironment reaches its limits and the pH of the tumor microenvironment becomes acidic. For instance, the extracellular pH (pHe) in the vicinity of normal differentiated cells is around 7.4, while the pH in the TME ranges between pH 5.5 and 7.4 [151,152].

**Figure 2:**
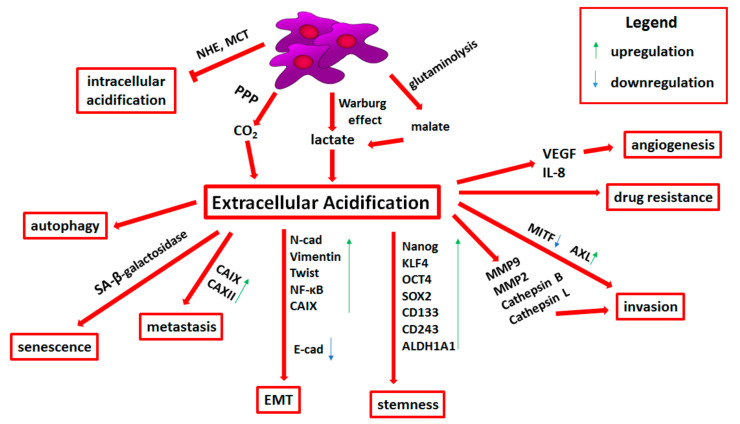
Influence of extracellular acidification on melanoma progression. Detailed descriptions can be found in the text. Abbreviations: NHE, Na/H exchanger; MCT, monocarboxylate transporters; PPP, pentose phosphate pathway; CA, carbonic anhydrase; N-cad, N-cadherin; NF-κB, nuclear factor-kappa B; E-cad, E-cadherin; KLF4, Krüppel-like factor 4; OCT4, octamer-binding transcription factor 4; SOX2, SRY-box transcription factor 2; ALDH, aldehyde dehydrogenase; MMP, matrix metalloproteinase; MITF, microphthalmia-associated transcription factor; VEGF, vascular endothelial growth factor; IL-8, interleukin 8; EMT, epithelial-to-mesenchymal transition.

There are several reasons responsible for this phenomenon. Firstly, cancer cells undergo the metabolic shift related to an increased level of glycolysis, even in the presence of oxygen, which is called “aerobic glycolysis”. The final product of glycolysis, pyruvate, is then reduced to lactate and exported to the extracellular space, thus leading to the TME acidification (Figure 2) [153,154].

Carbon dioxide (CO_2_), produced via the pentose phosphate pathway (PPP), highly active in tumor cells, also contributes to the acidification of the tumor niche (Figure 2). PPP produces one molecule of CO_2_ per one molecule of glucose. CO_2_ can be then hydrated and converted to hydrogen protons (H^+^) and bicarbonate by carbonic anhydrases (CA). This family of enzymes in humans consists of 16 isoforms. For instance, CAII acts intracellularly, while CAIX and CAXII extracellularly. H^+^ ions produced by CAs then function as acids [155].

Glutaminolysis is another catabolic process enhancing extracellular acidification (Figure 2). It involves the metabolic reactions of the tricarboxylic acid cycle and the malate-aspartate shuttle. Glutamine is converted to glutamate and then used to produce α-ketoglutarate. α-KG completes the TCA cycle in the mitochondria and undergoes conversion to malate. Following its export to the cytosol, malate is then decarboxylated to pyruvate and reduced to lactate, which, as mentioned above, contributes to the acidification of the tumor microenvironment [151,156]. The enzyme essential for lactic fermentation is lactate dehydrogenase (LDH). Its high level is correlated with the poor prognosis of advanced melanoma patients. This enzyme also constitutes a negative predictor of response to anticancer therapy [157].

Finally, acid uptake by skin fibroblasts has been shown to participate in maintaining the pH of the TME in a range favorable to tumor growth. The H^+^ ions extruded by cancer cells can be taken up by surrounding fibroblasts and directly transported between them through connexin-43-containing gap junctions. This phenomenon, known as “acid venting” prevents excessive extracellular acidification in solid tumors. Acid uptake by dermal fibroblasts is stimulated by TGFβ1 [158]. Moreover, Shu et al. have shown that melanoma-derived exosomes contain miR-155 and miR-210, which influence human adult dermal fibroblasts and lead to their metabolic reprogramming manifested by an increase in aerobic glycolysis, reduction of OXPHOS, and thus higher extracellular acidification [159].

Another miR affecting melanoma glycolysis is miR-216a-5p, which inhibits hexokinase 2 (HK2)—an enzyme initiating glycolysis. Reduction in HK2 activity results in a decrease of lactate level and acceleration of mitochondrial oxidation. Ectopic overexpression of miR-216-5p in melanoma cells led to a decreased cell proliferation rate. Moreover, a negative correlation between expression of miR-216a-5p and HK2 level in patients was observed, while low miR-216-5p level was associated with worse prognosis [160]

Yang et al. demonstrated the impact of miR-489-3p on melanoma proliferation, migration, and invasion. MiR-489-3p inhibits SIX1 (sine oculis homeobox 1), which is a main transcription factor modulating aerobic glycolysis. In melanoma patients’ samples, miR-489-3p level negatively correlated with SIX1 production, while its overexpression led to decreased proliferation, migration, invasion, and lactate production in vitro and reduced tumor volume in vivo [161]. Similar effect on extracellular acidification in melanoma has miR-150-5p, which similarly to miR-489-3p, inhibits SIX1, and thus reduces aerobic glycolysis. In patient-derived samples exhibiting elevated glycolysis, the expression of these miRs was diminished [162].

### 3.2. Transporters Related to pH Regulation

To avoid intracellular acidification, cells increase the expression or activity of transporters and pH-regulating channels [151]. The protons produced inside the cells are removed from the cytosol by transport proteins such as the Na/H exchanger (NHE), intensifying the acidification of the tumor niche (Figure 2) [152]. Vahle et al. indicated that NHE1 is strongly involved in the maintenance of intracellular pH homeostasis in melanoma [163]. Moreover, mutated BRAF-induced ERK stimulation intensifies phosphorylation and activation of NHE1. BRAF-mutated melanoma cells demonstrated increased intracellular pH dependent on elevated NHE1 activity [164]. In comparison with wild type BRAF, BRAF-mutated cells showed raised extracellular lactate concentrations [165]. On the other hand, Wahl et al. showed that lactate is extruded not by NHE, but mainly by monocarboxylate transporters MCT1 and MCT4, which transport lactate together with H^+^ ion in the same direction. Culturing melanoma cells under acidic conditions (pH 6.7) increased the activity of MCT1, confirming their role in extracellular pH regulation (Figure 2) [166].

Research has shown that acidification within the tumor microenvironment may be associated with a more aggressive melanoma phenotype [167]. It was indicated that under the influence of acidic pHe melanoma cells showed increased secretion of proteinases and proangiogenic factors as well as elevated invasive abilities and more effective suppression of the immune system [168,169,170]. Moreover, when they were inoculated into mice, these melanoma cells exhibited increased potential to form pulmonary metastases [169]. Pinheiro et al. also demonstrated that MCT4 and GLUT1 expression was significantly upregulated in metastatic samples of melanoma and correlated with poor patients’ prognosis. Furthermore, expression of MCT1 and MCT4 was associated with shorter overall survival [171].

The previously described phenomena of hypoxia and acidification are closely related. HIF molecule contributes to the Warburg effect, increasing the activity of LDH-A, which converts pyruvate to lactic acid, thus acidifying the TME [172]. Moreover, it has been shown that both oxygen deficiency and extracellular acidification induce the expression of genes encoding CAIX and CAXII [173,174]. Expression of these enzymes is associated with higher risk of metastasis and worse overall survival among melanoma patients (Figure 2) [175,176]. Andreucci et al. demonstrated that CAIX inhibitor—FC16-670A prevents upregulation of this metalloenzyme expression under acidosis, promoting apoptosis and necrosis only in acidified cancer cells [177]. Targeting CAIX seems to affect mainly acidic tumor cells exhibiting a highly malignant phenotype and high resistance to apoptosis. This form of treatment could represent a possible therapeutic approach to reduce tumor relapse [177]. Giuntini et al. indicated that CAXII inhibitor is also able to reduce invasive abilities of melanoma cells, which was associated with reduced phosphorylation of FAK kinase and the activity of MMP9 [176].

### 3.3. Acid-Resistant Phenotype of Melanoma Cells

In the case of melanoma, adaptation of cells to low pH and selection of cells showing an acid-resistant phenotype was observed. During this process, a significant part of the cells dies, however, acid-resistant cells also appear and after re-acclimatization to physiological pH, show even greater invasive abilities. These cells also exhibit the upregulation of signaling pathways important for tissue remodeling, cell cycle control, and proliferation [178]. It was also indicated that acid-resistant cells, which then migrated to regions of physiological pH, may more likely form metastasis [151]. Moreover, melanoma cells cultured in vitro under acidic conditions exhibit a remodeled phospholipids patterns. These cells produce longer and more unsaturated acyl chains compared to their counterparts cultured in neutral pH. This process was mediated by upregulation of genes involved in acyl chain desaturation, elongation, and transfer to phospholipids. Changes in lipid composition are able to influence the membrane dynamics and activity of proteins regulating the pH balance between the intracellular and extracellular environments [53].

Another adaptation mechanism to the acidic environment relies on autophagy (Figure 2). Marino et al. showed that human melanoma cells grown under acidic conditions induce autophagy and reduce the absorption of nutrients such as glucose and leucine. Moreover, inhibition of autophagy through the *ATG5* (autophagy related 5) knockdown reduced melanoma cell survival in low pH conditions [151,179]. Prolonged autophagy induced by nutrients deprivation, together with hypoxic conditions, may eventually lead to cell death. Matsuo et al. showed the importance of tumor microenvironment acidification mediated by lactic acid in promoting the survival of B16F10 mouse melanoma cells subjected to stress conditions [180].

### 3.4. Epithelial-to-Mesenchymal Transition and Stemness in Acid-Adapted Melanoma Cells

Exposure to acidic conditions is also able to promote epithelial-to-mesenchymal transition in melanoma cells. The acidic pH leads to a significant increase in the expression of EMT-related mesenchymal proteins such as N-cadherin, vimentin, and transcription factors—Twist and NF-kB, as well as results in E-cadherin downregulation (Figure 2) [181]. Moreover, it was demonstrated that acid-adapted melanoma cells co-express CD133, a known stemness marker, and CAIX to a greater extent compared to control cells. The latter one is a critical mediator of the self-renewal and invasive potential of cancer cells via transcriptional regulation of genes associated with EMT and stemness [182]. Melanoma cells cultured long-term in an acidic environment also demonstrated the induction of self-renewal and pluripotency markers (e.g., Nanog, KLF4 (Krüppel-like factor 4), OCT4 (octamer-binding transcription factor 4), and SOX2) as well as the overexpression of the cancer stem cells markers (e.g., CD133, CD243, and ALDH1A1) (Figure 2). This stem-like phenotype was reversible—when cells were retransferred to standard pH, their self-renewal capacity and expression of stem-related markers was decreased [182].

### 3.5. Extracellular Acidification-Induced Adhesion, Invasion, and Angiogenesis

Acidity of the TME also affects the adhesion, migration, and invasion of cancer cells. In melanoma, several molecules such as integrins α_2_β_1_, α_5_β_1_, or α_V_β_3_ are pH-dependent [183,184]. Stock et al. demonstrated that extracellular pH affects the migration of human melanoma cells by modulating the interplay between cells and ECM. Migration is inhibited, when the interaction is too strong in acidic or too weak in alkaline pHe [185].

It was also shown that the melanoma cells migration depends on the activity of NHE, which is strongly involved in the extracellular pH regulation. Its inhibition impaired melanoma cell motility [185]. Acidosis may promote the stability of focal adhesions through the stimulation of integrin-collagen binding in migratory membrane protrusions localized on the leading edge of the cell, so called lamellipodia [186]. NHE1 activity and low cell surface pH strengthen the cell-matrix interaction of melanoma cells at focal adhesions by modulating α_2_β_1_ integrin conformation [187]. At the same time, increased NHE1 activity may impair adhesion between cancer cells, thereby facilitating their dissemination [188].

NHE is not evenly expressed on the surface of human melanoma cells. It mainly localizes on the leading edge of the lamellipodium structure. As a result, the front surface of the cell membrane has acidic pH, while the back of the cell is more alkaline. This pH polarization can facilitate adhesion to the matrix at the front and the breakdown of focal adhesions at the rear of the cell [188,189]. Vahle et al. also indicated that in the presence of the NHE1-specific inhibitor cariporide, invasion was reduced by approximately 50%, while adhesion was unaffected [163].

Intravenous microscopic analysis has shown that in vivo the tumor pHe is acidic and the regions with the lowest pH are associated with the highest invasive potential [190]. Extracellular acidosis also activates phospholipase D, ERK1/2, p38 MAPK, and NF-κB in metastatic mouse melanoma, which induces MMP9 expression in cancer cells [191]. Rofstad et al. showed that human melanoma cells grown in vitro at acidic pH secrete increased amounts of a number of proteases (e.g., MMP2, MMP9, cathepsin B, and cathepsin L) as well as proangiogenic factors (i.e., VEGFA and IL-8) (Figure 2) [169]. It was also demonstrated that lactate secreted into the melanoma microenvironment is taken up by endothelial cells via MCT1, where it induces HIF-1α, and thus leads to the upregulation of growth factors associated with angiogenesis including VEGFR2 and bFGF (basic fibroblast growth factor) [192].

### 3.6. Drug Resistance Related to Tumor Niche Acidification

The acidic microenvironment can significantly affect the effectiveness of anticancer therapies, for instance, by influencing the distribution and absorption of slightly alkaline chemotherapeutic drugs, which leads to therapy resistance (Figure 2). This resistance is based on the “ion trap” model, taking into account the fact that weakly basic chemotherapeutic drugs accumulate in more acidic compartments. The acidic pHe can effectively impede weakly basic drugs from reaching their intracellular target, thereby reducing their cytotoxicity [151,193]. Moreover, following acidic exposure of melanoma cells, their increased resistance to anoikis was observed [24,25]. It was also indicated that BRAF V600E-mutated melanoma cells exposed to acidosis exhibited vemurafenib resistance, even though phosphorylated ERK was suppressed. In contrast, these cells were more sensitive to mTOR pathway inhibition than control cells, which suggests that acidification-induced melanoma phenotype exhibits preference towards mTOR rather than ERK signaling [194].

The TME acidosis also induces a senescent-like phenotype in melanoma cells characterized by induction of senescence-associated β-galactosidase activity, enlarged cell body size, cellular multinucleation, G_1_/G_0_ cell cycle arrest as well as inhibited cell proliferation (Figure 2). Transfer of acidosis-treated melanoma cells to physiological pH resulted in restoration of proliferation ability, even to a greater extent compared to previous conditions. Acidosis also led to enhanced resistance of melanoma cells to etoposide, a compound targeting actively dividing cells. This type of agent is not able to affect the acidosis-induced slow-proliferating cells, which results in drug resistance and may be responsible for tumor recurrence [167].

Moreover, in melanoma cell lines, extracellular acidosis induces upregulation of AXL receptor tyrosine kinase and reduction in MITF expression, which leads to the appearance of MITF^low^/AXL^high^ phenotype (Figure 2). Low MITF level is associated with invasive and senescent-like phenotype, which exhibits resistance to MAPK pathway inhibitors [167]. Recently, it was shown that melanoma cells use senescence to generate tumor-initiating cells with stem cell-like properties and to acquire chemotherapy resistance [167,195].

### 3.7. Immune Escape Mediated by Tumor Niche Acidification

Extracellular lactic acid is also able to influence the activity of immune cells. It can inhibit the antitumor effects of cytotoxic T cells and NK cells as well as reduce lymphocyte proliferation and maturation of dendritic cells. These immunosuppressive effects are not due to acidity per se, but rather a result of the lactic acid influx through the lactate/H^+^ co-transporter, which under neutral pH removes lactic acid from leukocytes [168,196,197]. It was demonstrated that high levels of melanoma cells-derived lactic acid present in the TME blocks the export of this molecule from T cells, thus disrupting their metabolism and function [198]. It also inhibits IFNγ production by CTLs [199].

Bohn et al. showed that intratumoral acidosis triggers cAMP-dependent signal transduction leading to the expression of the transcriptional repressor ICER (inducible cAMP early repressor) in tumor-associated macrophages, which induces their functional polarization toward a non-inflammatory M2 phenotype and promotes tumor growth [200].

Combination of bicarbonate, which neutralizes tumor acidity, with routinely used in melanoma anti-CTLA4 or anti-PD-1 therapy significantly improved the antitumor effects of these drugs in the murine melanoma model, which was resistant to bicarbonate monotherapy. Authors postulated that therapy based on bicarbonate acts by buffering the tumor pH to more neutral values, without affecting systemic pH [201]. This strategy was also used to improve cytotoxic T cell activity in patients with relapsed acute myeloid leukemia after allogeneic hematopoietic cell transplantation. Here, it was shown that sodium bicarbonate was responsible for T cell metabolic rescue, allowing for the incorporation of lactic acid in the TCA cycle as an additional energy source and thus increased IFNγ production [202].

Pilon-Thomas et al. also suggested that a similar effect could be achieved with the application of either isoform-specific CAIX or LDH inhibitors [201]. Chafe et al. confirmed this hypothesis. Expression of CAIX was associated with reduced immune system anticancer response of malignant melanoma patients. They also demonstrated that application of CAIX inhibitor combined with immune-checkpoint inhibitors led to the sensitization of melanoma to immune therapy. It was related to enhanced T helper cell response occurring as a result of the reduction of PD-1 expression on T cells present within the tumor niche [175].

### 3.8. Therapies Targeting Extracellular Acidification

There are some concepts of therapies directed against the acidity of the TME. One approach involves the development of weakly acidic compounds, which are able to enter the interior of solid tumors and release toxins in an acidic microenvironment [203]. Another therapeutic strategy is based on the inhibition of cellular proton pumps, which reduce the intracellular pH. This lowered pH within cells leads to decreased proliferation rate and induction of apoptosis in cancer cells. Milito et al. demonstrated that the proton pump inhibitor esomeprazole, activated by acidity, neutralizes tumor pH via inhibition of proton ejection. It led to the reduction of melanoma cells proliferation in vitro and promoted their apoptosis through caspase activation. Moreover, esomeprazole diminished melanoma tumor growth in mice and significantly increased the survival of examined animals, without any relevant side effects [204]. Administration of another proton pump inhibitor, omeprazole, significantly enhanced the effect of cisplatin in melanoma cells in mice, possibly due to the fact that cisplatin is a slightly alkaline drug, whose intracellular uptake is reduced in acidic TME [205].

## 4. Conclusions

The melanoma microenvironment is a very intricate structure, which influences the tumor progression and drug resistance. It consists of various stromal cells, i.e., keratinocytes, cancer-associated fibroblasts, adipocytes, and immune cells, connected through elements of extracellular matrix. The physicochemical aspects of the tumor niche such as acidity and oxygenation are also important for melanoma growth. Accurate and thorough knowledge concerning the vicinity of tumors, including melanoma, is critical to understand molecular basis of cancer progression and further develop novel therapeutic strategies. For maximum efficiency, the treatment has to focus not only on the elimination of cancer cells, but also has to consider the crosstalk between malignant cells and its niche. The data on the role of the features of tumor microenvironment such as hypoxia or extracellular acidification are very valuable, as they allow for better insight into the biology of melanoma cells. Processes like metabolism reprogramming, angiogenesis, stemness and cell plasticity, invasiveness, immune escape, and drug resistance are highly influenced by tumor niche oxygenation and acidification. Further development of hypoxia- and acidification-targeted therapies seems also valid, taking into account promising results obtained using in vitro and in vivo melanoma models.

## Figures and Tables

**Table 1:** A summary of Therapeutic Strategies Targeting the Hypoxic Melanoma Microenvironment.

The Therapeutic Approach	Treatment Description	Results and Utilized Research Model
Indirect targeting (angiogenesis)	Anti-VEGF antibodies and multikinase inhibitors	Phase I/II of clinical trials for novel drug combinations are still ongoing. Monotherapies were not effective, while combination treatment with chemotherapy induced short-term response [130].
	sVEGFR (vector or induced by GM-CSF)	Reduction of tumor angiogenesis, growth, and metastasis was observed in vitro and using murine melanoma model [131,132].
	Promotion of endothelial gap junctions (Sac-1004)	In murine melanoma model, vasculature normalization, inhibition of metastasis and EMT, reduction of cancer stem-like cells population was noted [133].
Direct targeting (HIF-1α inhibitors)	Acriflavine	Inhibitors blocked the metabolism and proliferation of melanoma cells in vitro [134].
	2-methoxyestradiol	Drug resensitized radioresistant cells and partially suppressed their glycolytic state [135].
	Cinnamaldehyde	Treatment reduced the invasiveness of melanoma cells in vitro and in vivo [136].
	Arylsulfonamide (64B)	The compound decreased the growth and metastasis of murine uveal melanoma [8].
Direct targeting (alleviation of hypoxic conditions)	Metformin (inhibition of oxygen consumption)	Improvement of anti-PD-L1 therapy was observed in murine melanoma model [137].
	Liposomes with hemoglobin and doxorubicin, followed by radiotherapy	Treatment efficiently blocked migration in vitro and inhibited tumor growth in mice [138].
	Multimodal platforms (CAT@aPDL1-SSL, mZDC, Au@MTM-HA)	Treated mice exhibited improved tumor infiltration by cytotoxic T cells, prolonged survival, and reduced metastasis rate [139,140,141].
Direct targeting (oxygen-sensitive treatment)	Prodrugs activated in hypoxia (tirapazamine, TH-302)	Combination therapy resulted in effective tumor size reduction in mice [142,143].
	Bacteria as drug delivery vectors (*S. typhimurium*) or oncolytic inducers (*C. novyi-NT*)	Bacteria-delivered vector reduced the proliferation of melanoma cells, while the oncolytic strain efficiently targeted solid tumors [144,145].

VEGF, vascular endothelial growth factor; sVEGFR, soluble vascular endothelial growth factor receptor; GM-CSF, granulocyte-macrophage colony-stimulating factor; EMT, epithelial-to-mesenchymal transition; HIF-1α, hypoxia-inducible factor 1α; PD-L1, programmed cell death ligand 1.

## Data Availability

Data sharing not applicable.
